# Novel dopamine receptor 3 antagonists inhibit the growth of primary and temozolomide resistant glioblastoma cells

**DOI:** 10.1371/journal.pone.0250649

**Published:** 2021-05-04

**Authors:** Sarah E. Williford, Catherine J. Libby, Adetokunbo Ayokanmbi, Arphaxad Otamias, Juan J. Gordillo, Emily R. Gordon, Sara J. Cooper, Matthew Redmann, Yanjie Li, Corinne Griguer, Jianhua Zhang, Marek Napierala, Subramaniam Ananthan, Anita B. Hjelmeland

**Affiliations:** 1 Department of Cell, Developmental and Integrative Biology, University of Alabama at Birmingham, Birmingham, AL, United States of America; 2 HudsonAlpha Institute for Biotechnology, Huntsville, AL, United States of America; 3 Department of Pathology, University of Alabama at Birmingham, Birmingham, AL, United States of America; 4 Department of Biochemistry and Molecular Genetics, University of Alabama at Birmingham, Birmingham, AL, United States of America; 5 Department of Radiation Oncology, University of Iowa, Iowa City, IA, United States of America; 6 Chemistry Department, Southern Research, Birmingham, AL, United States of America; Sechenov First Medical University, RUSSIAN FEDERATION

## Abstract

Treatment for the lethal primary adult brain tumor glioblastoma (GBM) includes the chemotherapy temozolomide (TMZ), but TMZ resistance is common and correlates with promoter methylation of the DNA repair enzyme O-6-methylguanine-DNA methyltransferase (MGMT). To improve treatment of GBMs, including those resistant to TMZ, we explored the potential of targeting dopamine receptor signaling. We found that dopamine receptor 3 (DRD3) is expressed in GBM and is also a previously unexplored target for therapy. We identified novel antagonists of DRD3 that decreased the growth of GBM xenograft-derived neurosphere cultures with minimal toxicity against human astrocytes and/or induced pluripotent stem cell-derived neurons. Among a set of DRD3 antagonists, we identified two compounds, SRI-21979 and SRI-30052, that were brain penetrant and displayed a favorable therapeutic window analysis of The Cancer Genome Atlas data demonstrated that higher levels of DRD3 (but not DRD2 or DRD4) were associated with worse prognosis in primary, MGMT unmethylated tumors. These data suggested that DRD3 antagonists may remain efficacious in TMZ-resistant GBMs. Indeed, SRI-21979, but not haloperidol, significantly reduced the growth of TMZ-resistant GBM cells. Together our data suggest that DRD3 antagonist-based therapies may provide a novel therapeutic option for the treatment of GBM.

## Introduction

While primary malignant brain tumors remain relatively rare, glioblastoma (GBM, grade IV astrocytoma) is the most common in adults [1]. The prognosis for GBM patients is very poor: standard of care results in a median survival of approximately 14.6 months, but some subsets of patients can have improved survival. Standard of care includes chemotherapy with the DNA alkylating agent temozolomide (TMZ) [1, 2], and TMZ resistance is associated with the DNA repair enzyme O6-methylguanine–DNA methyltransferase (MGMT) [3–9]. Methylation of the MGMT promoter predicts improved TMZ response, but MGMT promoter methylation status does not always dictate TMZ sensitivity or the radiosensitizing potential of TMZ [10–14]. Thus, improving GBM treatment requires consideration of both MGMT-dependent and independent mechanisms contributing to therapeutic resistance.

In order to develop novel treatments for GBM and other brain tumors, there is renewed interest in repurposing of drugs known to cross the blood brain barrier, such as antipsychotics and anticonvulsants [15, 16]. The neurotransmitter dopamine binds to G protein-coupled receptors to modulate many neurological processes including pleasure responses, learning, and motor control. The D2-like family of dopamine receptors includes dopamine receptor 2, 3, and 4 (DRD2, DRD3, and DRD4). Dysregulation of DRD2-mediated neurotransmission is implicated in many neurological disorders including addiction, Parkinson’s disease, schizophrenia, and attention-deficit hyperactivity disorder [17, 18]. Recent data also indicate a role for D2-like family members in GBM [19–22]. The DRD2 inverse agonist and antipsychotic haloperidol improved the efficacy of an epidermal growth factor receptor inhibitor, although haloperidol (which also has efficacy in the nanomolar range on DRD3, DRD4, and several other receptors) was insufficient to decrease tumor growth alone [22]. Another study suggested the importance of DRD4 for the regulation of GBM autophagy and demonstrated benefits for using the D4 antagonist L741742 [20]. Very few studies to date have investigated the expression of DRD3 in cancer, much less defined DRD3 function or effects of inhibition in GBM or other cancers.

Although DRD2 antagonists are common antipsychotics, the drugs have a large number of side effects including dystonia, dry mouth, and hypotension [23]. DRD2 and DRD4 are also widely expressed throughout the brain, and thus, based on the restricted expression of DRD3 to the nucleus accumbens and islands of Calleja in normal brain, our group and others have selected DRD3 as a target for development of novel anti-addiction therapies [24–28]. In an effort to develop novel DRD3 selective antagonist ligands, Dr. Ananthan’s laboratory synthesized and screened a library of approximately 300 compounds. Evaluations for binding affinity at DRD3, DRD2, and DRD4 receptors using standard radioligand displacement assays and membranes prepared from cells expressing DRD2 family members and functional antagonist activity determinations using a beta-arrestin recruitment assay led to the identification of several DRD3-selective compounds [29, 30]. Using a selected set of compounds from this group, our study sought to identify a specific anti-GBM DRD3 antagonist that could be effective alone or used in combinational therapies to increase the efficacy of the current standard of care. Based on the findings presented here, we believe that DRD3 antagonists could remain effective in TMZ-resistant and recurrent GBMs while limiting the undesirable side effects associated with current DRD2 family antagonists.

## Materials and methods

### Ethics statement

All applicable international, national, and/or institutional guidelines for the care and use of animals were followed. This article does not contain any studies with human participants performed by any of the authors. Passage of all patient-derived xenografts (PDXs) was approved through IACUC number 20255 at the University of Alabama at Birmingham.

### Cells and culture

GBM patient derived xenografts (PDX) D456 (Duke University), JX12, JX14, and JX39 (parental and TMZ resistant; Mayo Clinic), and 1016 (UAB) were propagated subcutaneously in athymic nude mice with assistance from the UAB Brain Tumor Bank or in the Hjelmeland laboratory in accordance with IACUC approval. U251 cells (parental and TMZ-resistant) were obtained and cultured as previously described [31]. Human astrocytes, mouse neurons and human neurons were purchased or generated as previously described [32–35]. PDXs were dissociated using the Worthington Biochem Papain Dissociation System (LK003150) and isolated GBM cells propagated as neurospheres in tumor initiating cell (TIC) media, to best model parental tumors [36]. Serum free TIC media was Gibco phenol red free DMEM/F12 containing Gem 21 (Gemini Biosciences), sodium pyruvate, penicillin/streptomycin, and 10 ng/mL of epidermal growth factor and fibroblast growth factor.

### Dopamine receptor basal level expression

GBM cells were plated in TIC media at a density of 250,000 cells per dish in T25 tissue culture flasks and cells collected after 24 hours. For immunoblotting, cells were lysed in RIPA buffer (Thermo Scientific 89901) supplemented to 2% SDS and proteins were separated to determine basal expression of Dopamine receptors 2, 3 and 4. Antibodies included those against Dopamine Receptor D2 (AB5084P, Millipore), Dopamine Receptor D3 (ab42114, Abcam), and Dopamine Receptor D4 (pAb-324405, Sigma).

### Cell growth assay

Cells were plated at a density of 1,000 (GBM, NHAs) or 80,000 (Neurons) cells per well in Corning black clear bottom 96 well plates. Cells were allowed to recover overnight and then treated with the indicated concentrations of antagonists or dimethyl sulfoxide (DMSO) as a vehicle control. Cells were incubated for a total of 7 days. A 1:1 ratio of Cell Titer Glo 2.0 (Promega) was then added to the plate, incubated for 15 minutes, and luminescence was read on a Synergy H1 (Bio Tek) microplate reader.

### Blood brain barrier penetration capacity

Concentrations of inhibitors in the blood and in the brain were analyzed by Pharmaron (Germantown, MD), following a cassette dosing protocol similar to those previously reported [37, 38].

### RNA sequencing

Prior to RNA extraction, samples were treated with proteinase K (Norgen). The extraction of RNA was then completed with the Norgen Total RNA Purification kit including the DNase kit per manufacturer’s instructions (Norgen). Briefly, 400 ul of RL Buffer with 1% beta-mercaptoethanol was added and then a homogenized cell suspension obtained. RNA integrity numbers, RIN, were measured using BioAnalyzer (Agilent) and ranged from (9.7–10 RIN). RNA was quantified using Qubit 2.0 Fluorometer (Thermofisher). Yields for RNA from cell pellets ranged from 368–2340 ng/ul. We used 1000 ng of total RNA as input to the NEBNext Poly(A) mRNA Magnetic Isolation Module (NEB). PolyA depleted RNA was used as input to the NEBNext Ultra RNA Library Prep Kit for Illumina (NEB). Libraries were barcoded using the NEBNext Multiplex Oligos for Illumina (NEB). We pooled all samples for sequencing on a NextSeq, with paired-end 75 base pair reads and sequenced an average of 6 million reads per sample with an average Q30 score of 96.39%. Samples were processed using our published primary analysis tool, aRNApipe [39]. Differential expression was determined using DESeq2 [40]. These data are available on the gene expression omnibus (GEO) under the accession number GSE148740.

### Statistical analysis

Statistics were performed with Graphpad Prism Version 7 (Graphpad Software Inc., La Jolla, CA) with resulting p-values indicated in the figure legends. In general, one-way ANOVA was performed with the Tukey test.

## Results

### DRD3 antagonists significantly inhibit the growth of GBM cells and cross the blood brain barrier

**Fig 1:**
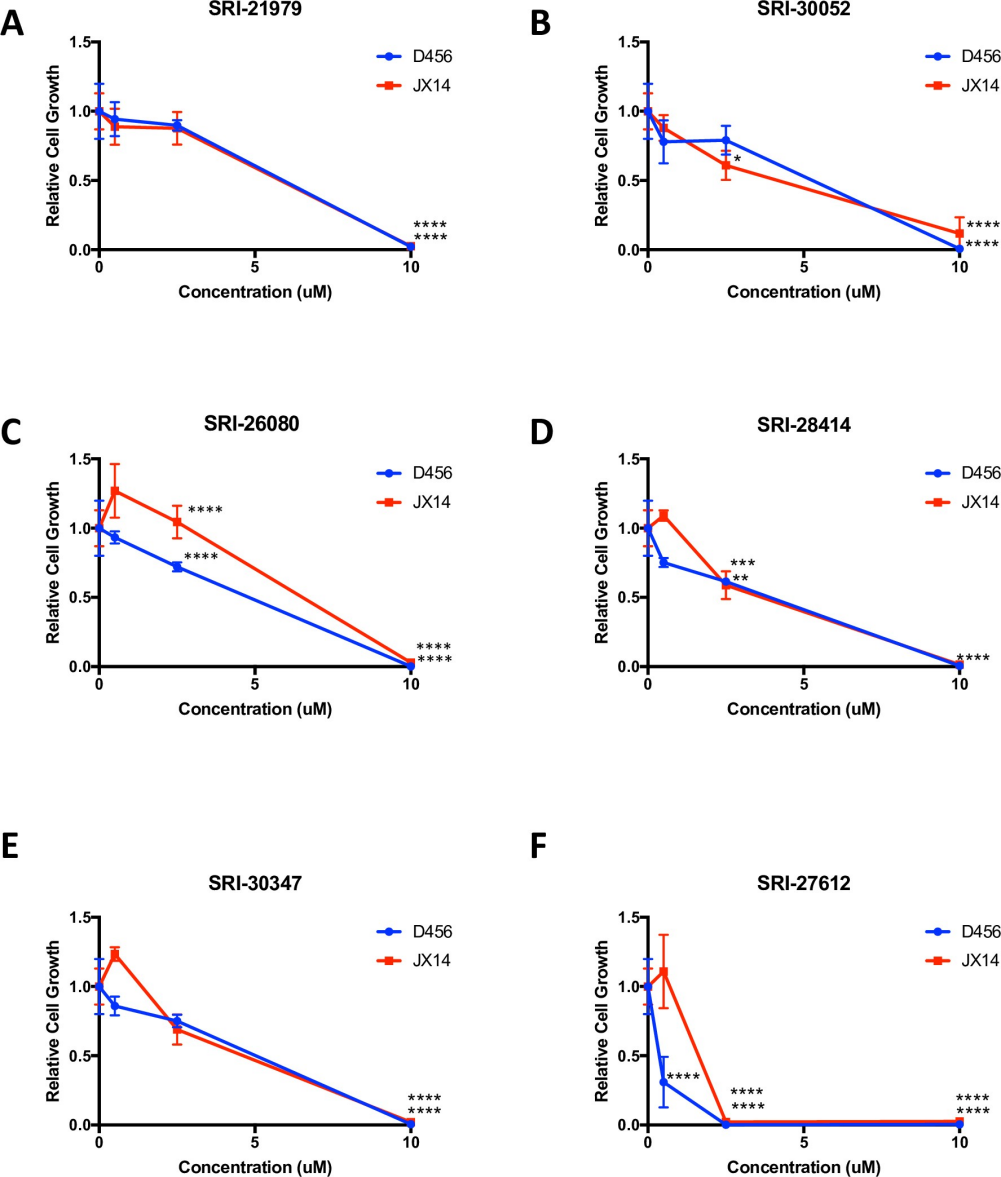
DRD3 antagonists significantly inhibit the growth of GBM PDX-derived neurospheres. Cells isolated from JX14 (red) or D456 (blue) GBM PDXs were treated with vehicle control or the indicated concentrations of (A) SRI-21979, (B) SRI-30052, (C) SRI-26080, (D) SRI-28414, (E) SRI-30347, or (F) SRI-27612 and growth measured after seven days using Cell Titer assays. ****p≤ 0.0001, ***p<0.001, **p<0.01, *p<0.05 with ANOVA comparison to vehicle control.

**Fig 2:**
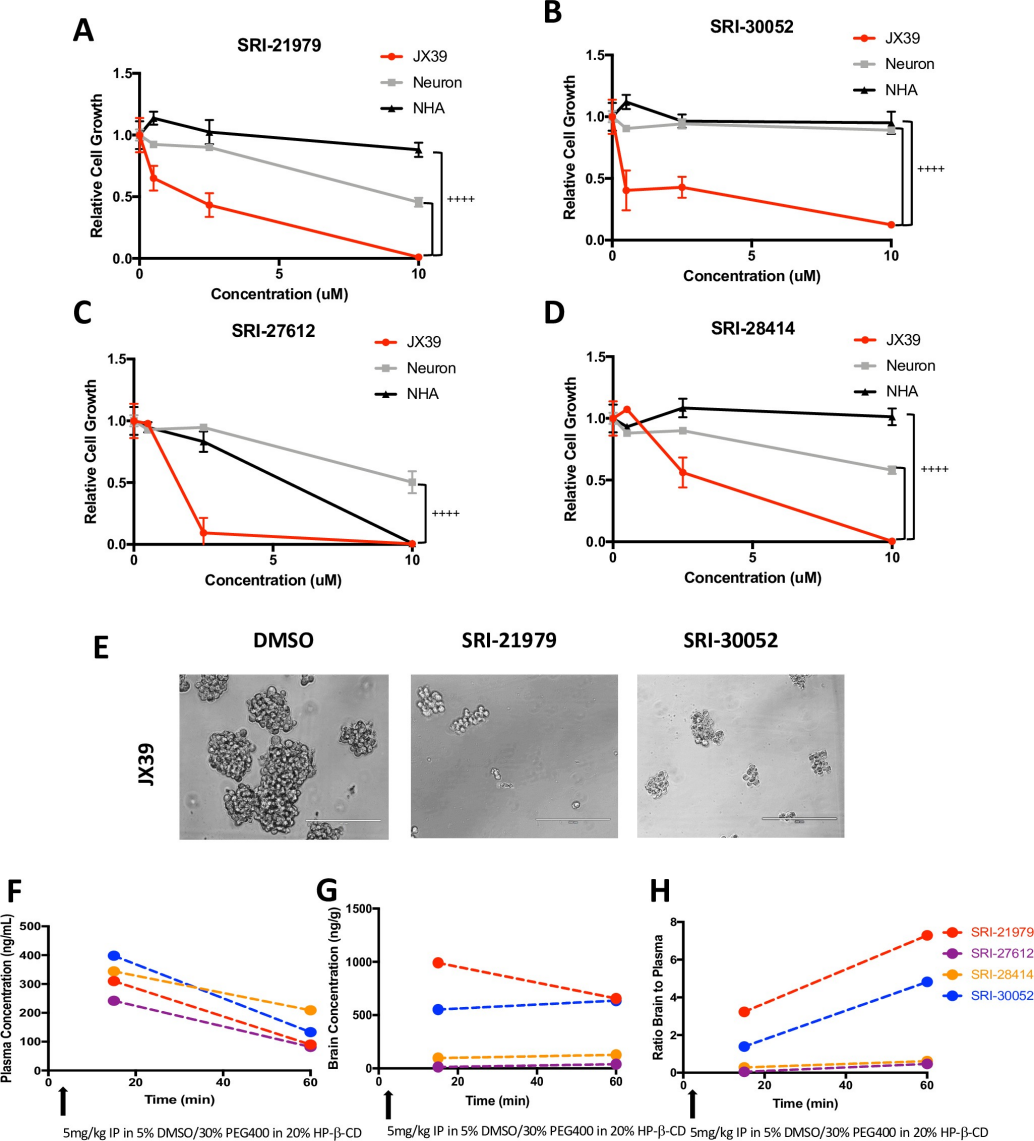
DRD3 antagonists have greater growth inhibitory potential against GBM cells than normal human astrocytes or neurons and cross the blood brain barrier. Cells isolated from JX39 (red) GBM PDXs, normal human astrocytes (NHA, black), and induced pluripotent stem cell derived neurons (grey) were treated with vehicle control or the indicated concentrations of **(A)** SRI-21979, **(B)** SRI-30052, **(C)** SRI-27612, or **(D)** SRI-28414. Growth in cell titer assays was determined seven days after treatment. Representative images of cells treated with SRI-21979 and SRI-30052 are shown **(E)**. Scale bar represents 200um. ++++p<0.0001 with ANOVA comparison of effects of 10 mM of the indicated drug in JX39 cells in comparison to NHAs and/or neurons. Levels of the indicated DRD3 antagonist detected via LC-MS in the **(F)** plasma or **(G)** brain at 15 or 60 minutes post intraperitoneal injection of 5 mg/kg drug with no detection in vehicle controls. **(H)** Brain to Plasma ratios for each DRD3 antagonist are shown. SRI-21979 (red) and SRI-30052 (blue) were found to accumulate in the brain relative to plasma but SRI-27612 (purple) and SRI-28414 (orange) were also detected in the brain. Time of injection is indicated with arrows.

To determine the potential of anti-GBM therapies targeting the DRD3 pathway, we first tested the GBM growth inhibitory capacity of a panel of 9 novel, selective DRD3-selective antagonists [29, 30]. The structures of these compounds and their binding affinities to DRD2 and DRD3 are provided in S1 Table. When GBM cells isolated from two different xenografts were treated with 10 μM of the DRD3 antagonists, three drugs (SRI-21459, SRI-21502, and SRI-21980) had minimal effects (S1 Fig). However, 10 μM of six of the DRD3 antagonists (SRI-21979, SRI-30052, SRI-26080, SRI-28414, SRI-30347, and SRI-27612) was sufficient to prevent GBM growth (Figs 1 and 2). Several of these compounds (SRI-21979, SRI-30052, SRI-27612, and SRI-28414) significantly decreased the growth of GBM cells derived from at least one of three different xenografts tested at concentrations less than 10 μM (Figs 1 and 2). For these 4 compounds, between 198 nM and 4.5 μM was sufficient to decrease growth of PDX-derived neurospheres by fifty percent. As expected, IC50s varied depending on the compound and the GBM cell type: IC50s for SRI-21979 ranged from 1.2 μM and 4.5 μM, SRI-30052 from 650 nM and 3.3 μM, SRI-27612 from 198 nM and 1.3 μM, and SRI-28414 from 2.2 μM and 3 μM. Considering these results, we next tested SRI-21979, SRI-30052, SRI-27612, and SRI-28414 in non-malignant astrocytes (NHAs) and IPSC-derived or mouse neurons to prioritize compounds displaying minimal toxicity to normal brain cells. Treatment of NHAs or neurons with several DRD3 antagonists had no growth inhibitory effects (Fig 2A–2D), and growth inhibition was significantly lower in non-tumorigenic cells in comparison to GBM cells for the majority of DRD3 antagonists tested (Fig 2). For example, the IC50 for SRI-21979 in NHAs was at least 20 fold greater than that in GBM cells and was at least 2 fold greater in neurons (Fig 2A). The IC50 for SRI-30052 was more than 50 fold higher in NHAs than in GBM cells, and we were unable to produce a growth inhibition at any of the concentrations tested for neurons (Fig 2B). In comparison to GBM cell IC50s for SRI-27612, IC50s were approximately 3 or 9 fold higher in NHAs or neurons respectively (Fig 2C). As for SRI-28414, the IC50 was more than 4 fold higher in neurons than GBM cells while none of the concentrations tested were able to decrease growth of astrocytes by fifty percent (Fig 2D). These results indicated that the novel DRD3 antagonists could have a high therapeutic index, due to their ability to target PDX-derived neurospheres without having a similar extent of growth inhibition in astrocytes or neurons. However, it is important to note that differences in standard cell culture conditions between PDX-derived GBM cells and non-neoplastic brain cells, such as media type and basal cell numbers plated per well, complicate a direct comparison of toxicity *in vitro*.

Based on our results suggesting the potential of SRI-21979, SRI-30052, SRI-27612, and SRI-28414 as anti-GBM therapies, these compounds were tested for their ability to cross the blood brain barrier (BBB) (Fig 2F–2H). All four DRD3 antagonists were detectable in the plasma and brains of mice at fifteen and sixty minutes after intraperitoneal injection (Fig 2F and 2G). However, levels of SRI-27612 and SRI-28414 in the brain were low and substantially less than that of SRI-21979 and SRI-30052. The brain-to-plasma ratio was highest for SRI-21979 followed by SRI-30052. Furthermore, the brain-to-plasma ratio for SRI-21979 and SRI-30052 increased over time (Fig 2H). These data indicate the ability of these DRD3 antagonists to be effective anti-GBM treatments: while the BBB is often compromised at the tumor site, drugs that penetrate into the non-tumor brain have a better potential to target GBM cells that have already escaped from the main tumor mass.

### DRD3 is expressed in primary and TMZ-resistant GBM cells and correlates with worse prognosis in MGMT unmethylated GBMs

In order to ensure the potential of DRD antagonism in GBM, we profiled the expression of the dopamine receptor 2 family members DRD2, DRD3, and DRD4 in PDX derived GBM cells (S2A–S2F Fig). Expression of DRD2 and DRD4 (S2A and S2C Fig) appeared higher than DRD3 (S2B Fig) in all samples tested, although there are limitations of making comparisons of protein expression with different antibodies within the context of immunoblotting. Quantification was performed, and each protein band was normalized to its respective actin band (S2D–S2F Fig). Overall, the level of each protein, DRD2 (S2D Fig), DRD3 (S2E Fig), and DRD4 (S2F Fig), is relatively similar across all of the xenolines tested (S2B–S2D Fig). We have also profiled expression of DRD2, 3, and 4 (S2G–S2I Fig) mRNA in GBM patient samples, and non-tumor samples. This data was obtained from The Cancer Genome Atlas, using Gliovis [41]. Both DRD2 and DRD4 have significantly higher expression in the non-tumor samples than the GBM samples (S2G and S2I Fig), while DRD3 has similar expression between GBM and non-tumor (S2H Fig). As DRD2 and 4 are more widely expressed than DRD3 in non-malignant brain and DRD2 antagonists have multiple side effects, the data support the potential for targeting DRD3 in GBM [30]. Western analysis further confirmed the presence of DRD3, as well as DRD2 and DRD4 in both parental and TMZ-resistant GBM cells (S3A–S3E Fig).

**Fig 3:**
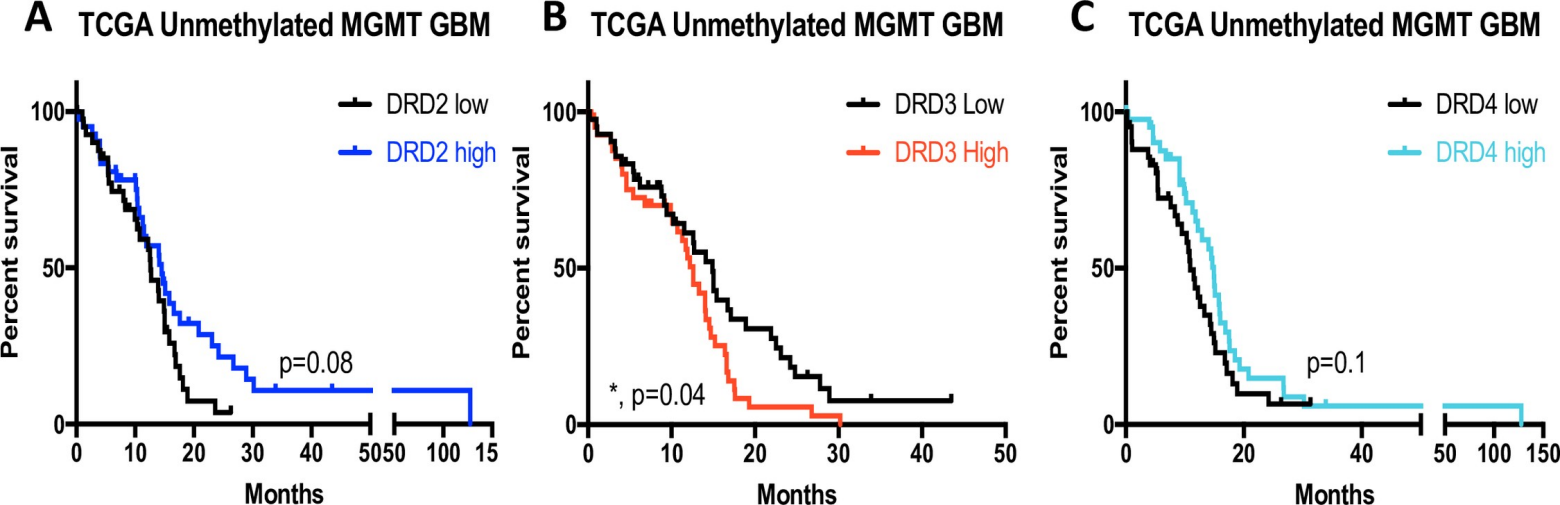
DRD3 correlates with worse prognosis in MGMT unmethylated GBMs. Survival of patients with primary, unmethylated MGMT GBMs segregated by **(A)** DRD2, **(B)** DRD3, or **(C)** DRD4 mRNA levels (highest or lowest 25%, n = 42). p values for Log-rank analysis are shown, data downloaded from GlioVis (http://gliovis.bioinfo.cnio.es).

The expression of DRD2-like family members and their correlations with patient outcomes were evaluated next in TCGA. We focused on the MGMT unmethylated GBMs, for which novel treatments are most urgently needed. We determined the association of DRD2-like family member expression with patient prognosis (Fig 3A–3C). We found that higher levels of DRD3 mRNA were associated with worse patient prognosis in primary MGMT unmethylated GBMs (Fig 3B). However, a similar effect was not observed when outcomes of patients with MGMT unmethylated GBMs were segregated based on DRD2 (Fig 3A) or DRD4 (Fig 3C) mRNA levels. These data suggest the potential for a specific effect or regulation of DRD3 in MGMT unmethylated (TMZ-resistant) tumors that is worthy of further investigation.

### SRI-21979 significantly decreases the growth of TMZ-resistant cells

To determine if DRD3 inhibition was capable of reducing the growth of TMZ-resistant GBM cells, U251 parental and TMZ-resistant (UTMZ) GBM cells as well as those isolated from a JX39 isogenic PDX pair were used. Resistance to TMZ in JX39T and UTMZ cells relative to the parental cells was confirmed (S4 Fig), and we previously published MGMT expression levels in the U251 cells [35].

**Fig 4:**
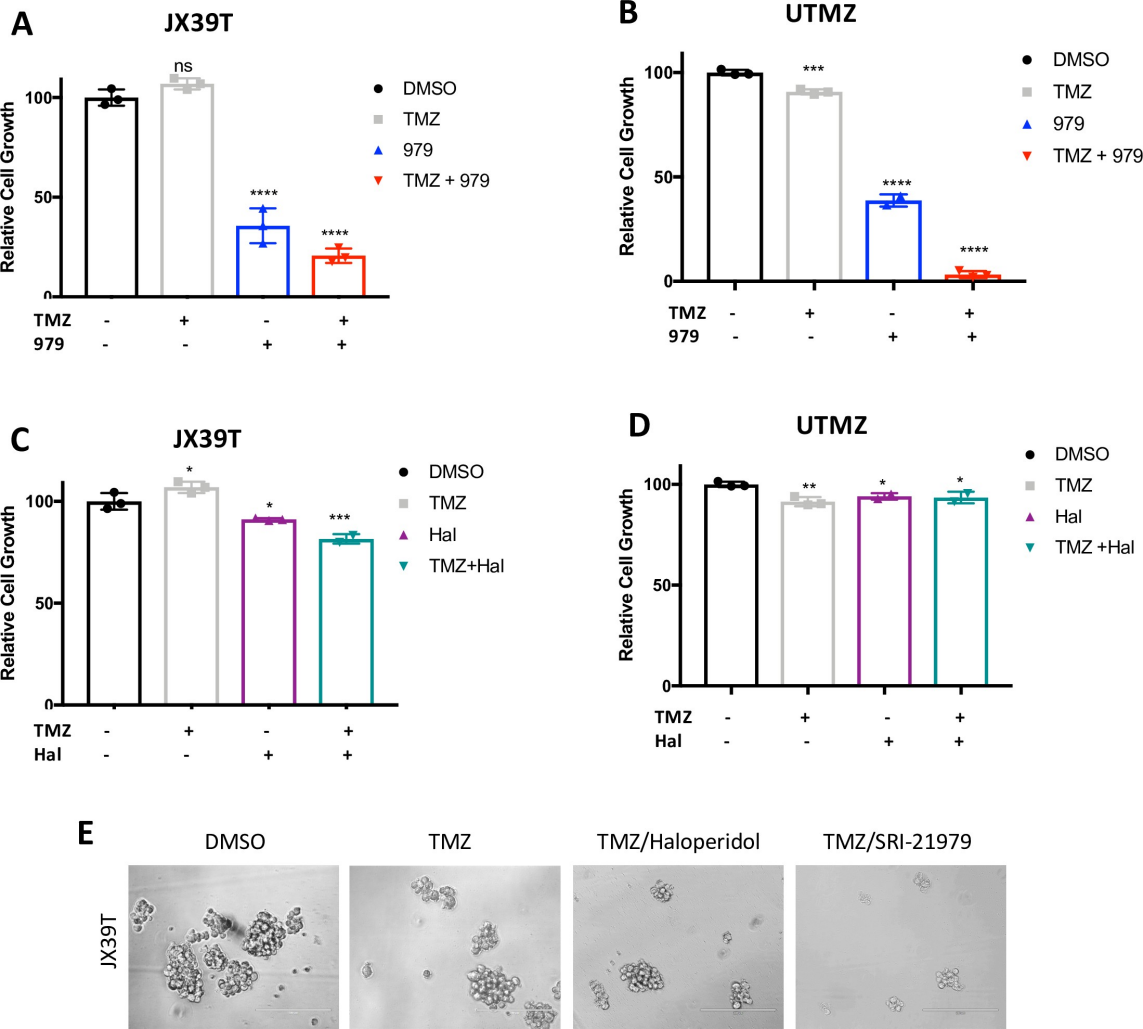
SRI-21979 shows efficacy in TMZ-resistant cells. JX39 (**A**) and U251 (**B**) TMZ-resistant cells (indicated as JX39T and UTMZ respectively) treated with vehicle, 100 mM TMZ, 5 mM SRI-21979, or TMZ and SRI-21979 as indicated. JX39T **(C)** and UTMZ (**D**) cells treated with vehicle, 100 mM TMZ, 5 mM haloperidol, or TMZ and haloperidol as indicated. (**E**) Representative images of cultures of JX39T cells treated with vehicle, TMZ, TMZ and haloperidol, or TMZ and SRI-21979 are shown. Scale bar represents 200um. **p<0.05, ***p<0.001, **** p≤ 0.0001 with ANOVA comparison to TMZ.

Monotherapy with increasing concentrations of SRI-21979 or haloperidol demonstrated that TMZ-resistant cells could remain growth inhibited by 10 μM of the compounds (S4 Fig). SRI-21979 alone reduced growth of JX39T and UTMZ GBM cells, and more substantial decreases were observed with addition of TMZ, (Fig 4A and 4B), whereas similar results were not observed with haloperidol (Fig 4C and 4D). Representative photos of JX39T cells validate the quantification by indicating minimal effects of addition of haloperidol to TMZ but decreased growth with the addition of SRI-21979 (Fig 4E). Overall, the data indicate that DRD3 antagonist-based therapies may remain effective in TMZ-resistant GBM for which novel therapies are urgently needed. The data suggest the possibility that DRD3 antagonists like SRI-21979 may increase the sensitivity of GBMs to TMZ, but additional studies would be needed to determine any additive or synergistic benefits. Importantly, these data do not demonstrate that DRD3 plays a direct role in promoting chemo-resistance in GBM, only that DRD3 may serve as a useful therapeutic target in chemoresistant and/or recurrent GBM, which is extremely difficult to treat.

### Treatment with SRI-21979 resulted in changes in transcript levels of several genes, including those involved in cAMP signaling and apoptosis

**Fig 5:**
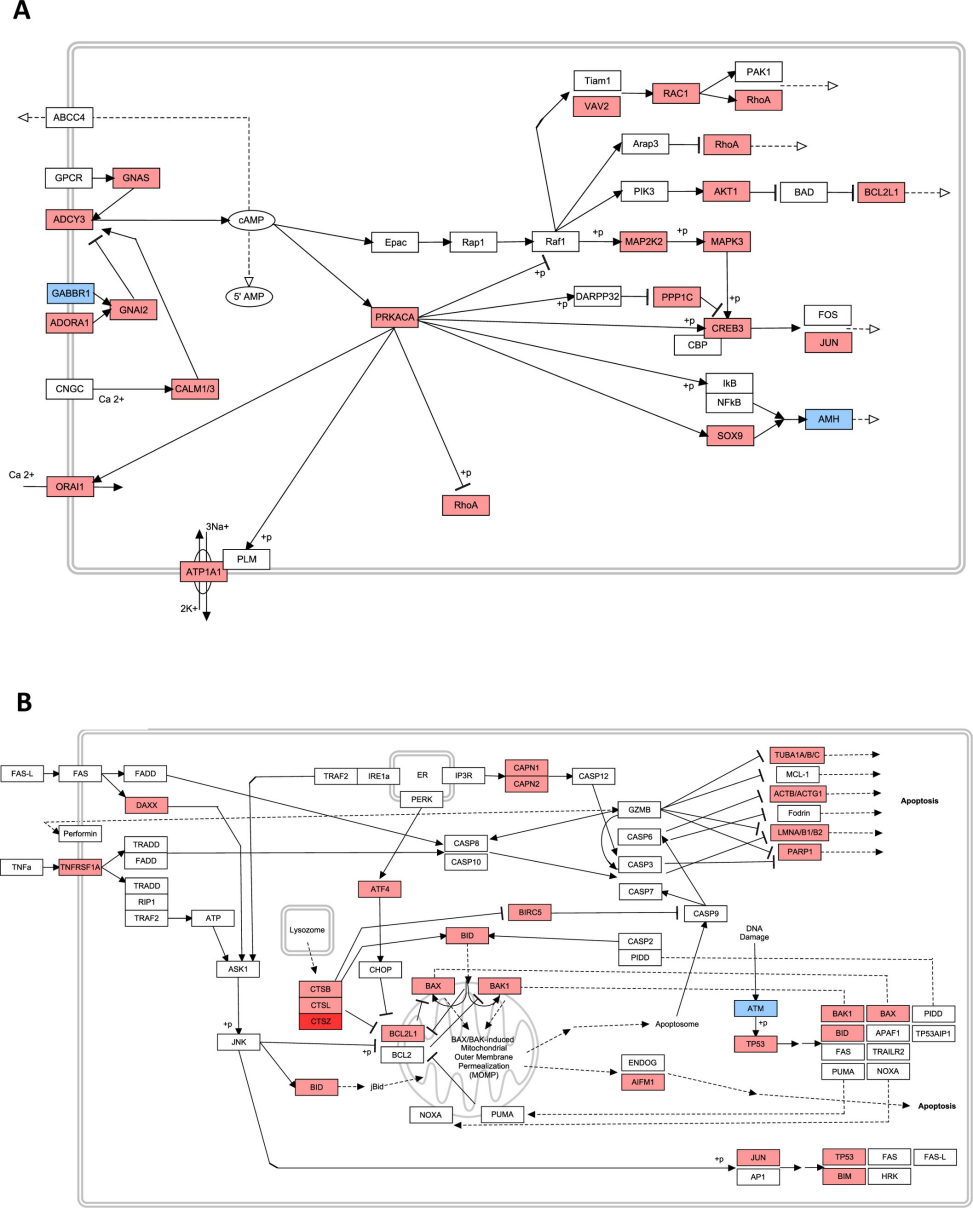
SRI-21979 alters the expression of transcripts involved in cAMP signaling and apoptosis. Pathway analysis of transcripts upregulated in cAMP signaling (**A**) and apoptosis (**B**), following treatment with SRI021979. Light red genes are a fold change >1, while blue indicates a fold change of <1. Dark red genes are those with a fold change >5. Transcripts shown in white, do not display any change between control and treatment groups.

To elucidate mechanisms of action for these compounds, RNA-sequencing was performed on GBM PDX-derived neurospheres treated for 24 hours with or without SRI-21979. This compound was chosen for additional molecular testing as it had the highest blood to plasma ratio and evidence for a high therapeutic index. Many of the transcripts differentially expressed between the vehicle (DMSO) treated PDX-derived neurospheres and those treated with SRI-21979 were involved with 3’, 5’-cyclic AMP (cAMP) signaling, with a large majority of the genes involved with this pathway showing increased transcript levels (Fig 5A). As D2-like receptors (including DRD3) inhibit adenylyl cyclase [42–44], which converts adenosine triphosphate to cAMP, increased cAMP signaling would be one predicted response of these inhibitors. Some cells increase cell death in response to elevated cAMP signaling [44–46], and we found that the apoptosis pathway contained many transcripts that were increased following treatment with SRI-21979 (Fig 5B).

## Discussion

Our data provides evidence that DRD3 is an anti-GBM target, including in chemotherapy-resistant tumors. Our finding that elevated DRD3 receptor expression correlates with worse survival in MGMT unmethylated patients (in whom TMZ is less effective), suggests that our *in vitro* data may have relevance for patient care. However, it is important to note that there are many ways through which GBMs are able to withstand chemotherapy. One such method may be the ability of these cells to reprogram their mitochondrial electron transport chain. The cells which undergo this reprograming effectively release lower levels of reactive oxygen species (ROS), and thus their apoptotic signaling is decreased [31, 47]. As UTMZ cells employ this mechanism of resistance and are sensitive to DRD3 antagonists, investigation of DRD3 antagonism in the recurrent setting could be broadly effective. Considering the inability of TMZ or any treatment to currently provide a GBM cure, our data provide important insights into strategies for DRD antagonism for GBM treatment, although additional studies directly assessing the role of DRD3 through shRNA or CRISPR based approaches are needed. In addition, our findings further support the repurposing of drugs designed to target brain signaling pathways; DRD3 antagonists have been primarily developed for other neurological disorders such as addiction.

While we have focused our study on GBM, a role for dopamine receptors has been implicated in other cancer types. Schizophrenic patients being treated with dopamine receptor antagonists had reduced incidence of several cancers [48], suggesting roles for DRD2-like family members in cancer development. Dopamine receptor antagonists primarily targeting DRD2 inhibited TIC growth in lung cancer [49] and leukemia [50]. Another group has also shown that increased activation of DRD2 in GBM cells leads to increased sphere-forming capacity, as well as increased metabolism [51], further identifying the D2-like family of receptors as a therapeutic target. Our preliminary review of data in cBioportal (cbioportal.org) indicated that a portion of neuroendocrine prostate (16.7%), cervical (4.5%), ovarian (>4%), and head and neck (>3%) cancers have amplifications of DRD3. These tumors may be sensitive to DRD3 inhibition, or, more broadly, D2-like receptor inhibition, as a strategy to elevate cAMP signaling to promote apoptosis similar to our RNA-sequencing findings. Thus, it is possible that DRD3 antagonism could provide a therapeutic option for other tumor types if amplification or aberrant cell signaling in multiple cancers results in DRD3 expression.

The restricted expression of DRD3 in the brain and non-tumorigenic adult tissues along with its expression in GBM cells suggests the potential for a large therapeutic window. Our studies demonstrated growth inhibitory effects of the tested DRD3 antagonists, which were minimal in NHAs or neurons. The variety of PDX lines shown to have significant growth reduction indicate that these treatments may be successful in all subsets of GBM. Additional studies outside the scope of the current manuscript are needed to define a maximum tolerated dose of the DRD3 antagonists. Once dosing is optimized, testing of the novel DRD3 antagonists would ideally be compared in parental and TMZ-resistant GBMs in combination with standard of care *in vivo*. Our *in vitro* data suggest that DRD3 antagonists may sensitize TMZ-resistant cells to TMZ, but additional studies would be needed to determine if there is any benefit of the DRD3 antagonists in vivo alone or in combination with standard of care. Whether improved survival could also be observed when tumor bearing mice were treated with other DRD antagonists that have efficacy against DRD3 should also be explored. As haloperidol treatment did not improve TMZ efficacy *in vitro* in our experiments, there may be distinct dopaminergic signals that need to be defined for increasing sensitivity to chemotherapy. Alternatively, the drugs or the strategy of DRD3 antagonism itself may only be effective in a specific subgroup of GBMs that has not yet been determined.

Of the DRD3 antagonists evaluated here, those that were able to decrease GBM growth are from the same family of urea-based compounds. This provides a useful backbone for further modifications: a more selective and potent drug with improved blood brain barrier permeability or oral bioavailability would be expected to provide a greater survival advantage for GBM bearing mice. However, we must consider the possibility that this class of drugs has as yet unknown off-target effects that contribute to their ability to inhibit GBM cell growth.

There is an urgent need for better treatments for GBM, and any therapy that could extend median survival or increase the percentage of long-term survivors could be important for patients. DRD3 antagonism, including with SRI-21979, showed promise against GBM in our studies. We believe that continuing to increase our understanding of the role of DRD receptors in GBM biology and elucidation of any differences in function or pattern of expression among D2-like family members is important for developing novel combinatorial therapies.

## Supporting information

S1 Table Structures, binding affinities (DRD2 and DRD3), and molecular weights of novel DRD3 antagonists tested for effects on GBM cell growth. (TIF)

S1 Fig Novel DRD3 antagonists for which 10 μM was not sufficient to reach an IC50. GBM cells isolated from D456 PDX xenografts were treated for seven days with up to 10 μM of the indicated DRD3 antagonists and results normalized to the vehicle control.
(TIF)

S2 Fig Expression of Dopamine Receptor 2 **(A),** 3 **(B),** and 4 **(C)**. Quantification of westerns via the ration of protein of interest to the corresponding loading control for DRD2 **(D)**, DRD3 **(E)**, and DRD4 **(F)**. Protein Lysates were collected from cells derived from five different GBM xenografts: D456, JX39, JX14, JX12, and 1016, as well as two non-neoplastic cells lines: Normal human astrocytes and neural progenitor. mRNA expression from patient samples was obtained from the TCGA for both non-tumor and GBM **(G-I)**. Protein was loaded at 40ug per lane for DRD3, and 20ug per lane for DRD2 and DRD4. **** p≤ 0.0001, **p<0.001, *p<0.05 unpaired t-test comparison.
(TIF)

S3 Fig (A) Expression of Dopamine Receptor 2, 3, and 4. Quantification of westerns via the ration of protein of interest to the corresponding loading control for DRD2 (B), DRD3 (C), and DRD4 (D). Lysates were collected from cells derived from parental (P) and TMZ-resistant (TMZ-R) JX39 xenografts or U251 cells. Protein was loaded at 40ug per lane for DRD3, and 20ug per lane for DRD2 and DRD4. **** p≤ 0.0001, **p<0.001, *p<0.05 unpaired t-test comparison.
(TIF)

S4 Fig Parental JX39P **(A)** and parental U251 **(B)** cells display sensitivity to TMZ, Haloperidol, and SRI-21979. TMZ-resistant JX39 **(**JX39T, **C)** and TMZ-resistant U251 **(**UTMZ, **D)** cells display resistance to TMZ, with sensitivity to Haloperidol and SRI-21979. DMSO was used as a vehicle control in the absence of drug. **** p≤ 0.0001, **p<0.001, *p<0.05 ANOVA comparison to vehicle control.
(TIF)

S1 Western blots (PDF)
