# Supplementary figures and images for: Novel dopamine receptor 3 antagonists inhibit the growth of primary and temozolomide resistant glioblastoma cells
Source: PLoS One. 2021 May 4;16(5):e0250649. doi: 10.1371/journal.pone.0250649 (PMC8096095; doi:10.1371/journal.pone.0250649)

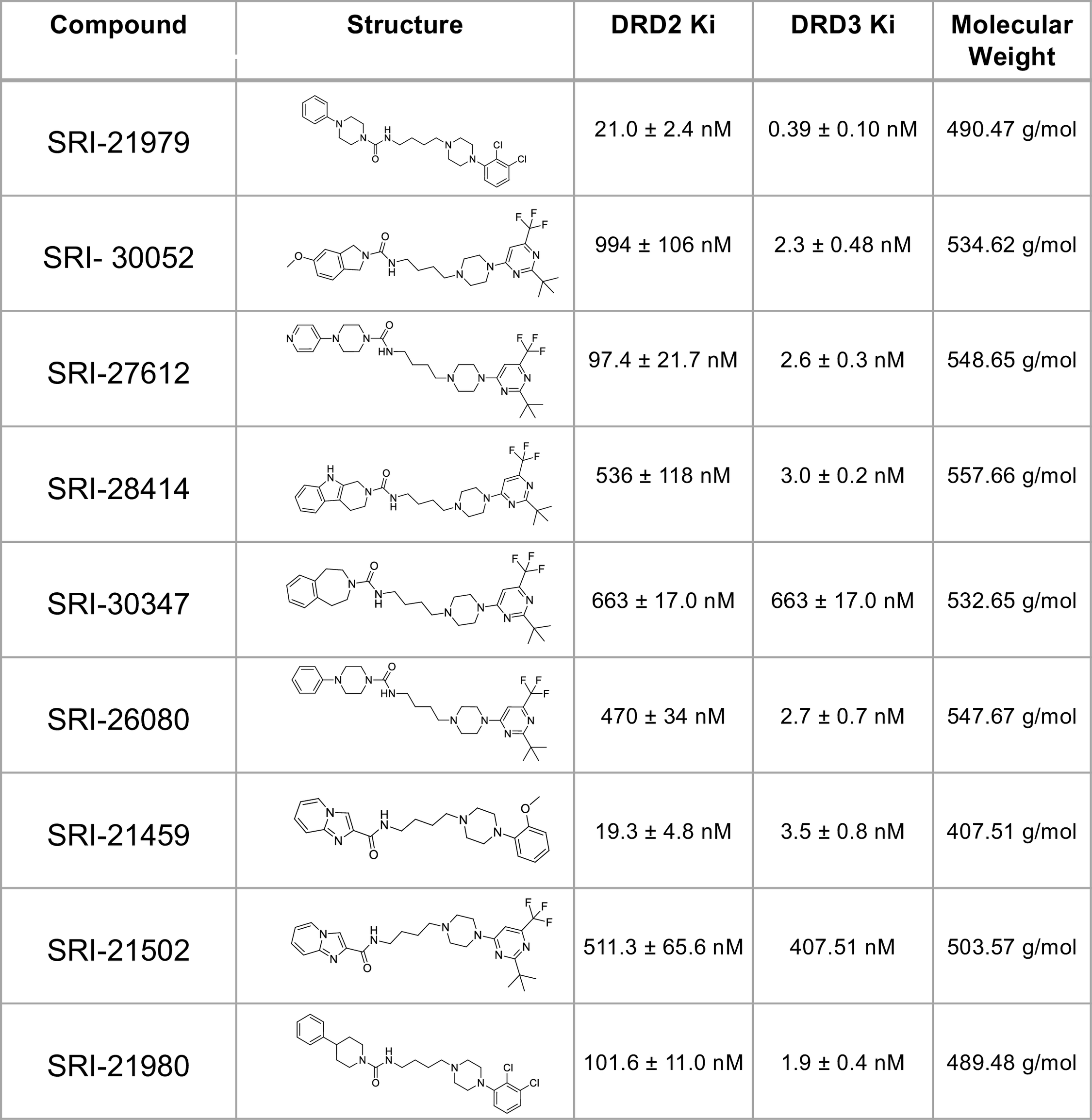

Supplement: S1 Table — (TIF) [file pone.0250649.s001.tif]

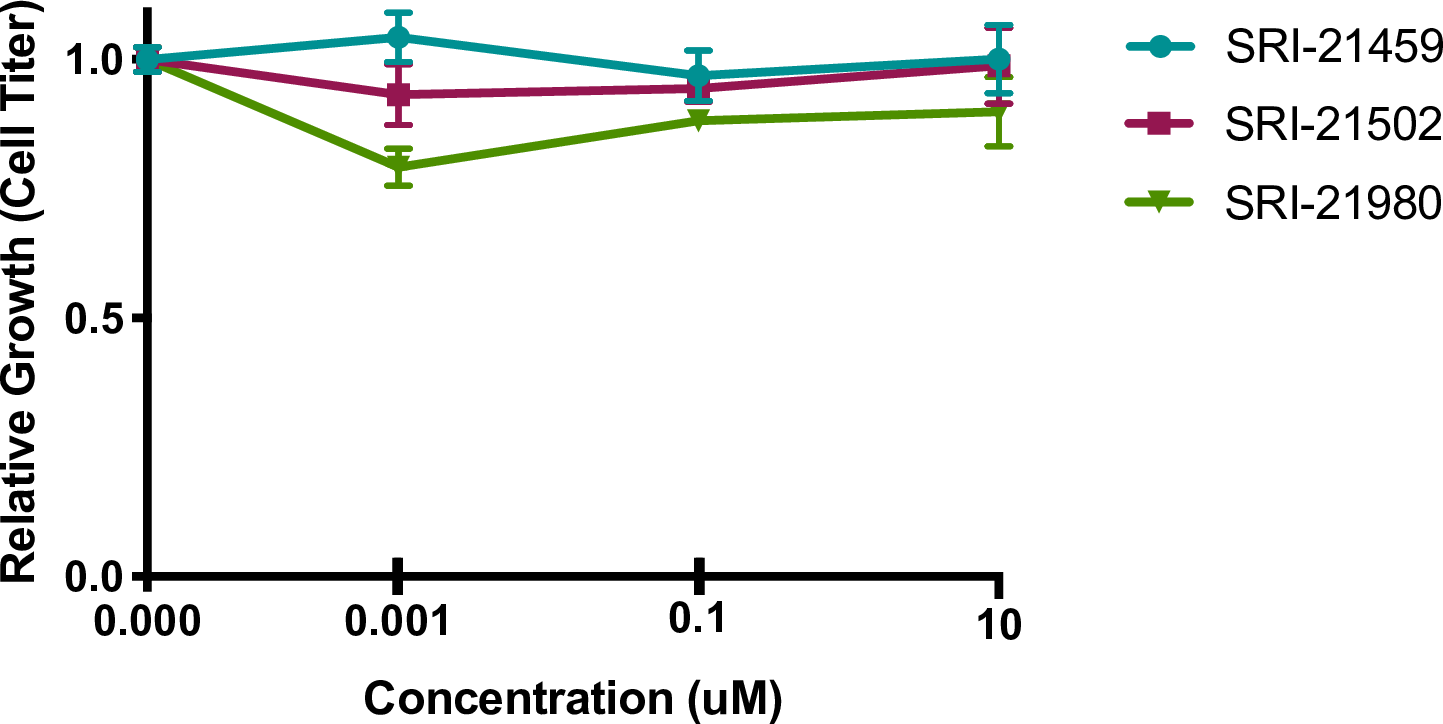

Supplement: S1 Fig — GBM cells isolated from D456 PDX xenografts were treated for seven days with up to 10 μM of the indicated DRD3 antagonists and results normalized to the vehicle control. (TIF) [file pone.0250649.s002.tif]

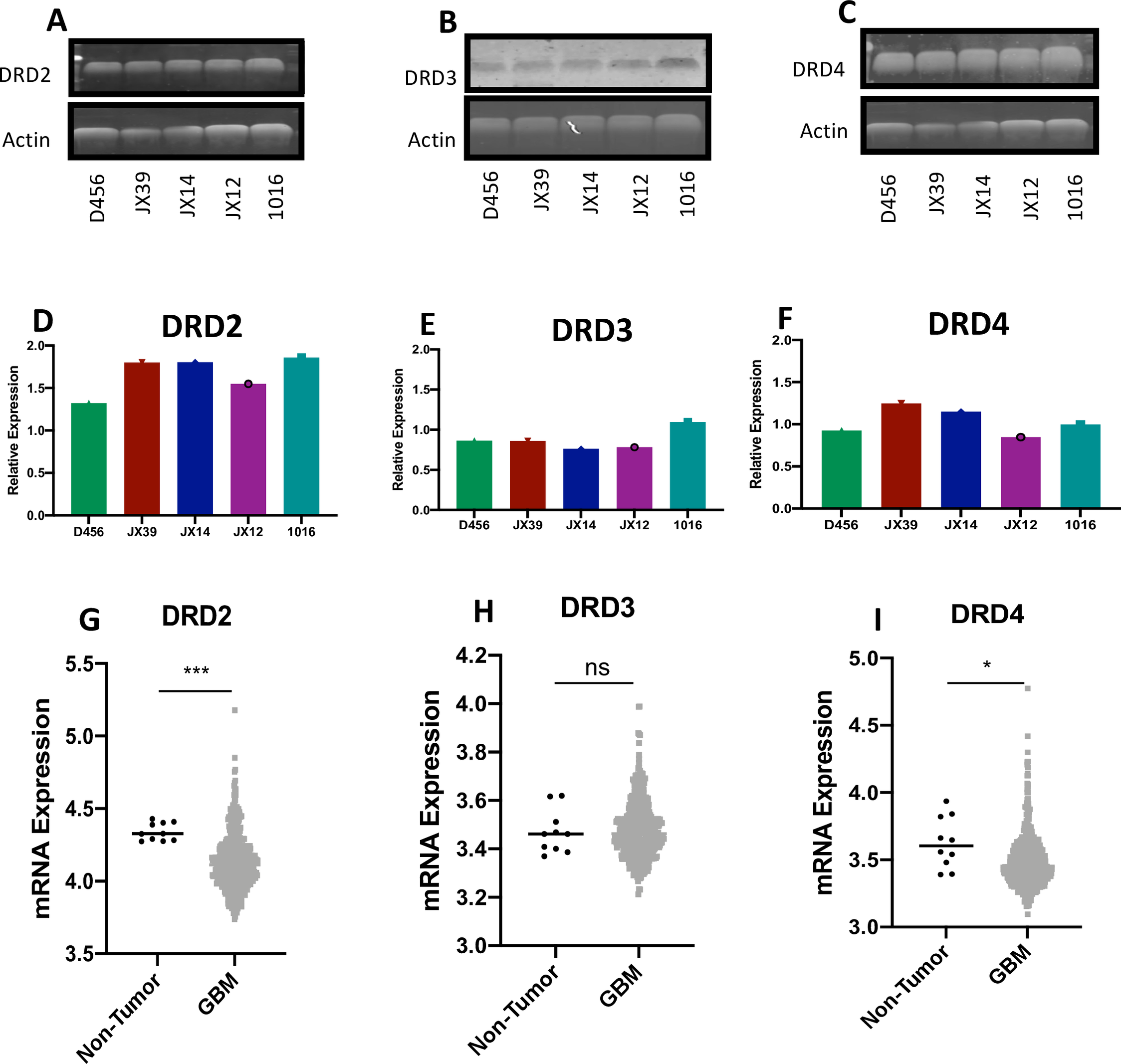

Supplement: S2 Fig — Expression of Dopamine Receptor 2 (A), 3 (B), and 4 (C). Quantification of westerns via the ration of protein of interest to the corresponding loading control for DRD2 (D), DRD3 (E), and DRD4 (F). Protein Lysates were collected from cells derived from five different GBM xenografts: D456, JX39, JX14, JX12, and 1016, as well as two non-neoplastic cells lines: Normal human astrocytes and neural progenitor. mRNA expression from patient samples was obtained from the TCGA for both non-tumor and GBM (G-I). Protein was loaded at 40ug per lane for DRD3, and 20ug per lane for DRD2 and DRD4. **** p≤ 0.0001, **p<0.001, *p<0.05 unpaired t-test comparison. (TIF) [file pone.0250649.s003.tif]

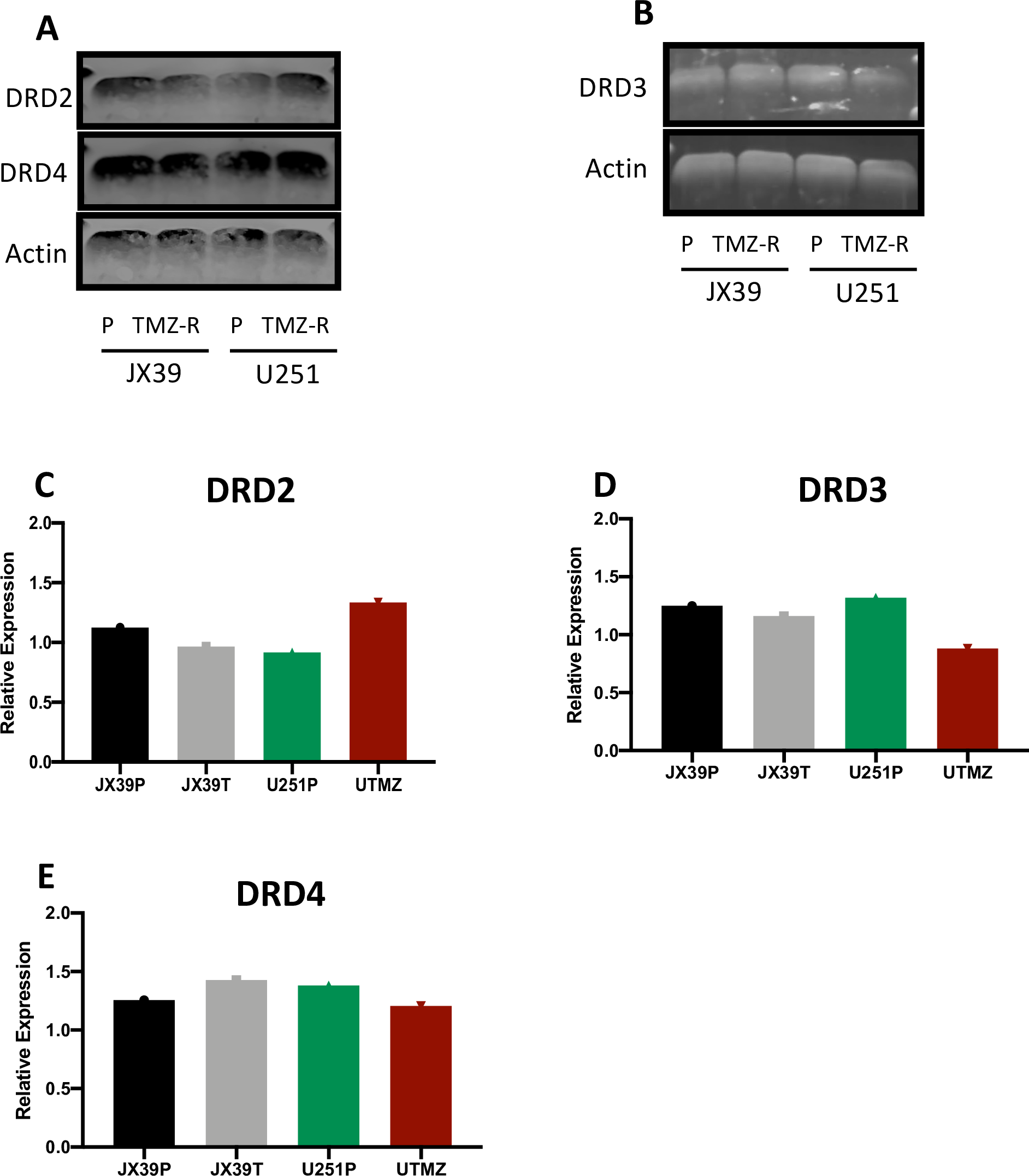

Supplement: S3 Fig — (A) Expression of Dopamine Receptor 2, 3, and 4. Quantification of westerns via the ration of protein of interest to the corresponding loading control for DRD2 (B), DRD3 (C), and DRD4 (D). Lysates were collected from cells derived from parental (P) and TMZ-resistant (TMZ-R) JX39 xenografts or U251 cells. Protein was loaded at 40ug per lane for DRD3, and 20ug per lane for DRD2 and DRD4. **** p≤ 0.0001, **p<0.001, *p<0.05 unpaired t-test comparison. (TIF) [file pone.0250649.s004.tif]

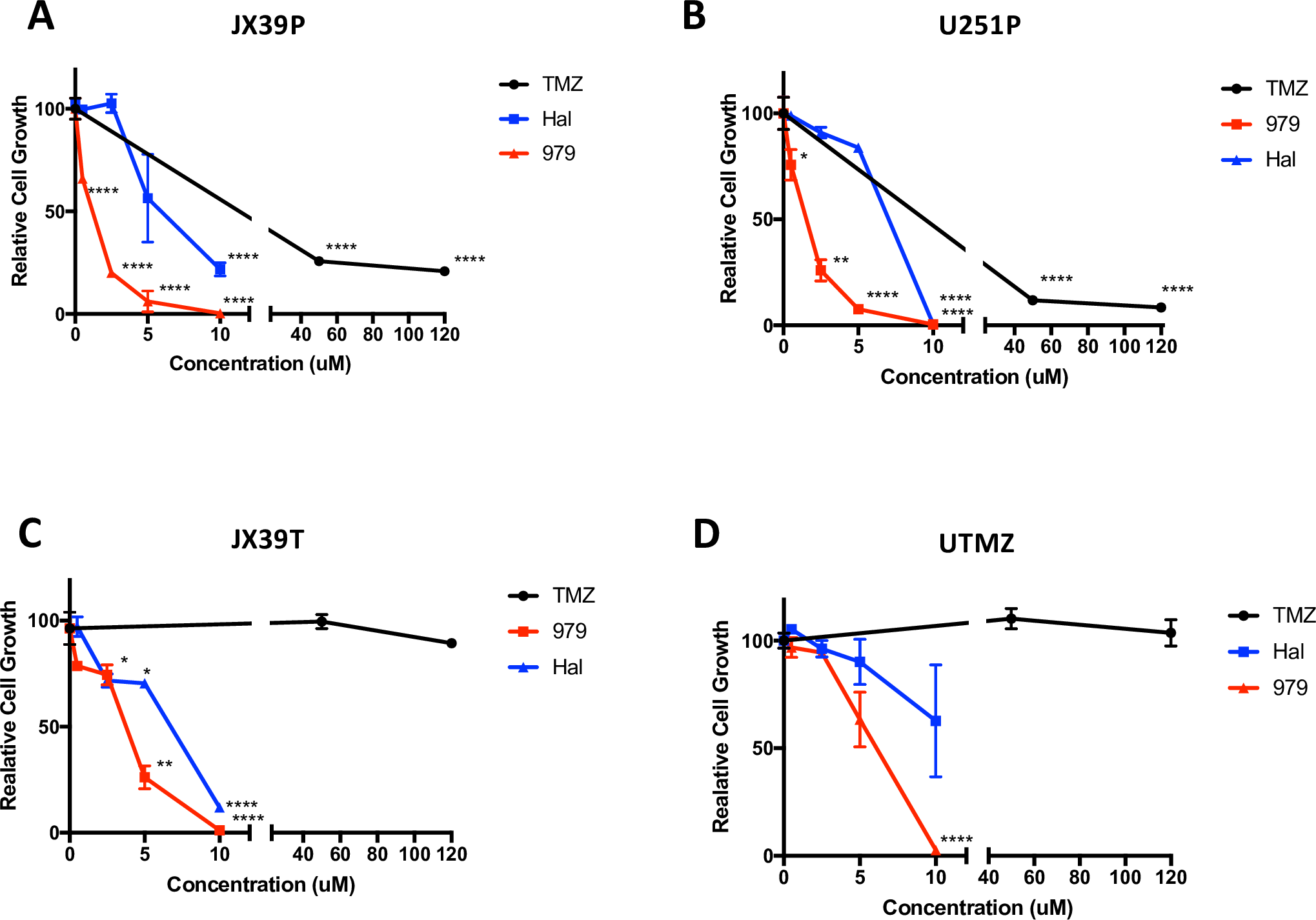

Supplement: S4 Fig — Parental JX39P (A) and parental U251 (B) cells display sensitivity to TMZ, Haloperidol, and SRI-21979. TMZ-resistant JX39 (JX39T, C) and TMZ-resistant U251 (UTMZ, D) cells display resistance to TMZ, with sensitivity to Haloperidol and SRI-21979. DMSO was used as a vehicle control in the absence of drug. **** p≤ 0.0001, **p<0.001, *p<0.05 ANOVA comparison to vehicle control. (TIF) [file pone.0250649.s005.tif]

DRD2 MW ~50Kda

MW:50

MW:50

JX39P

JX39T

U251P

U251T

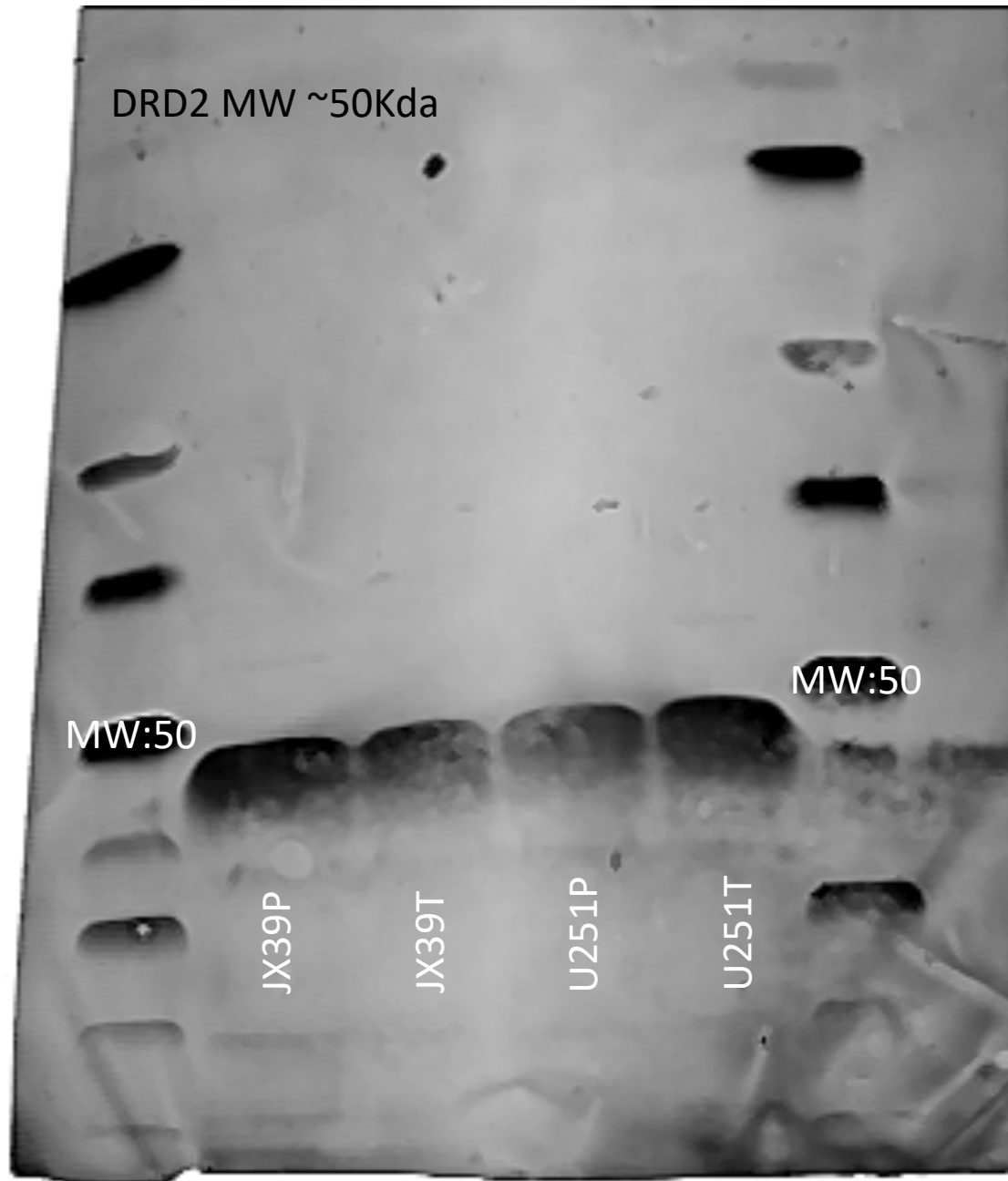

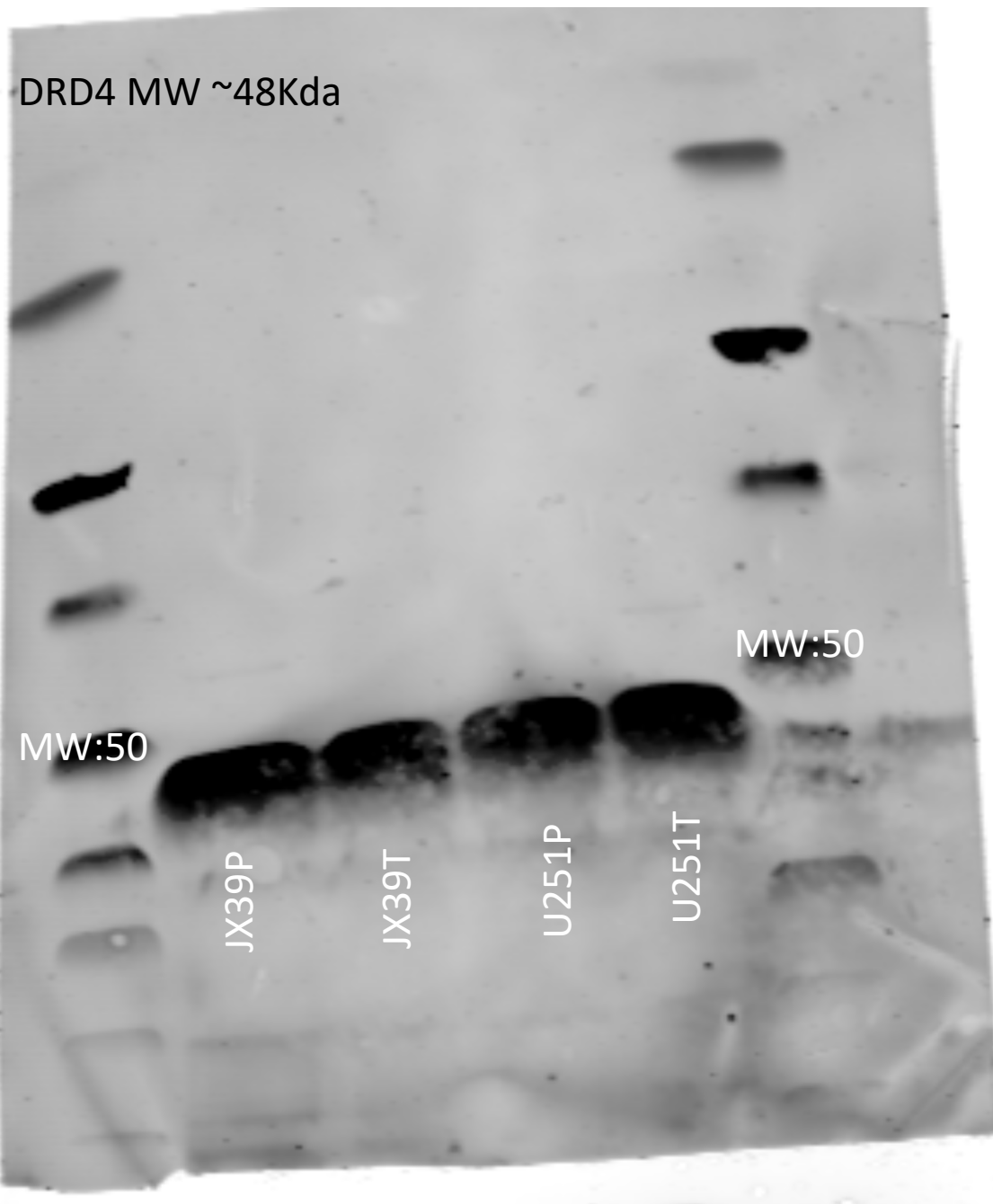

Actin (for DRD2 and DRD4) MW ~42Kda

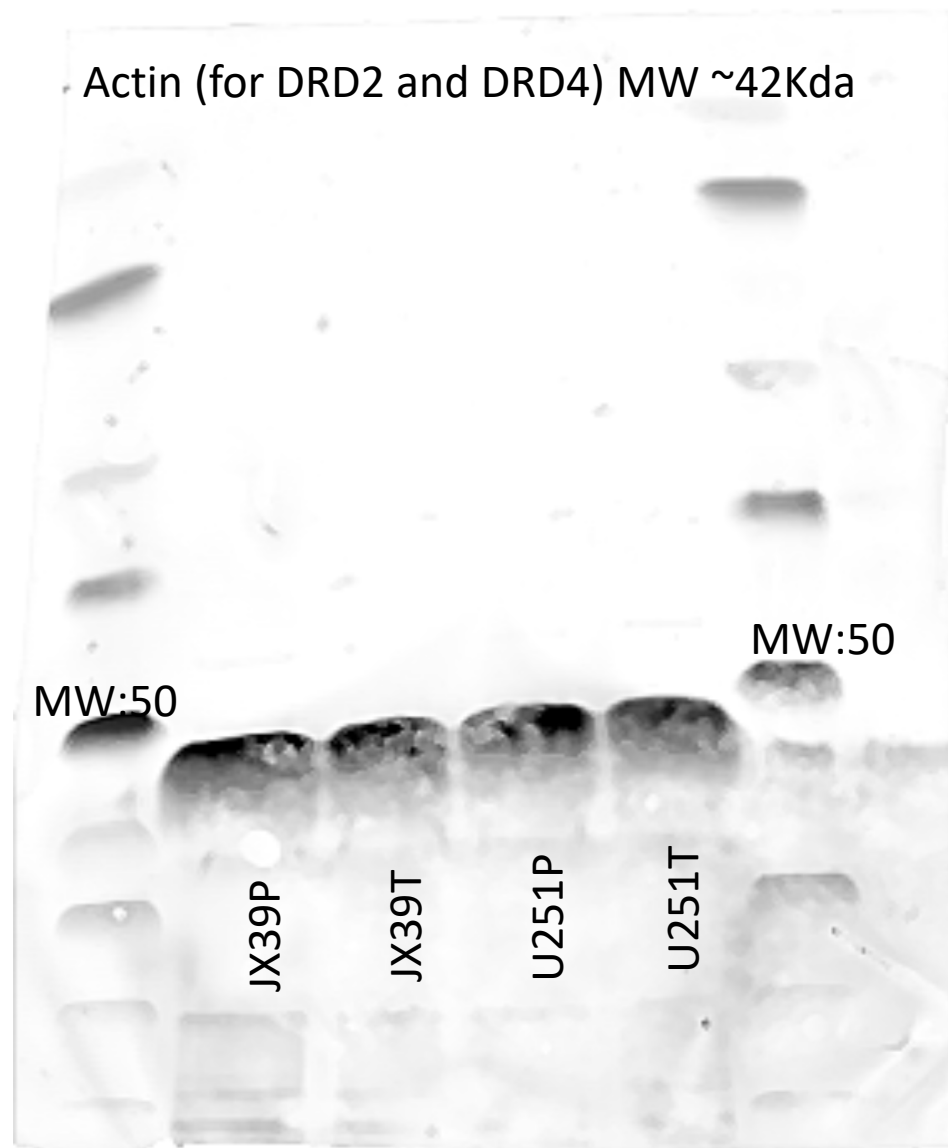

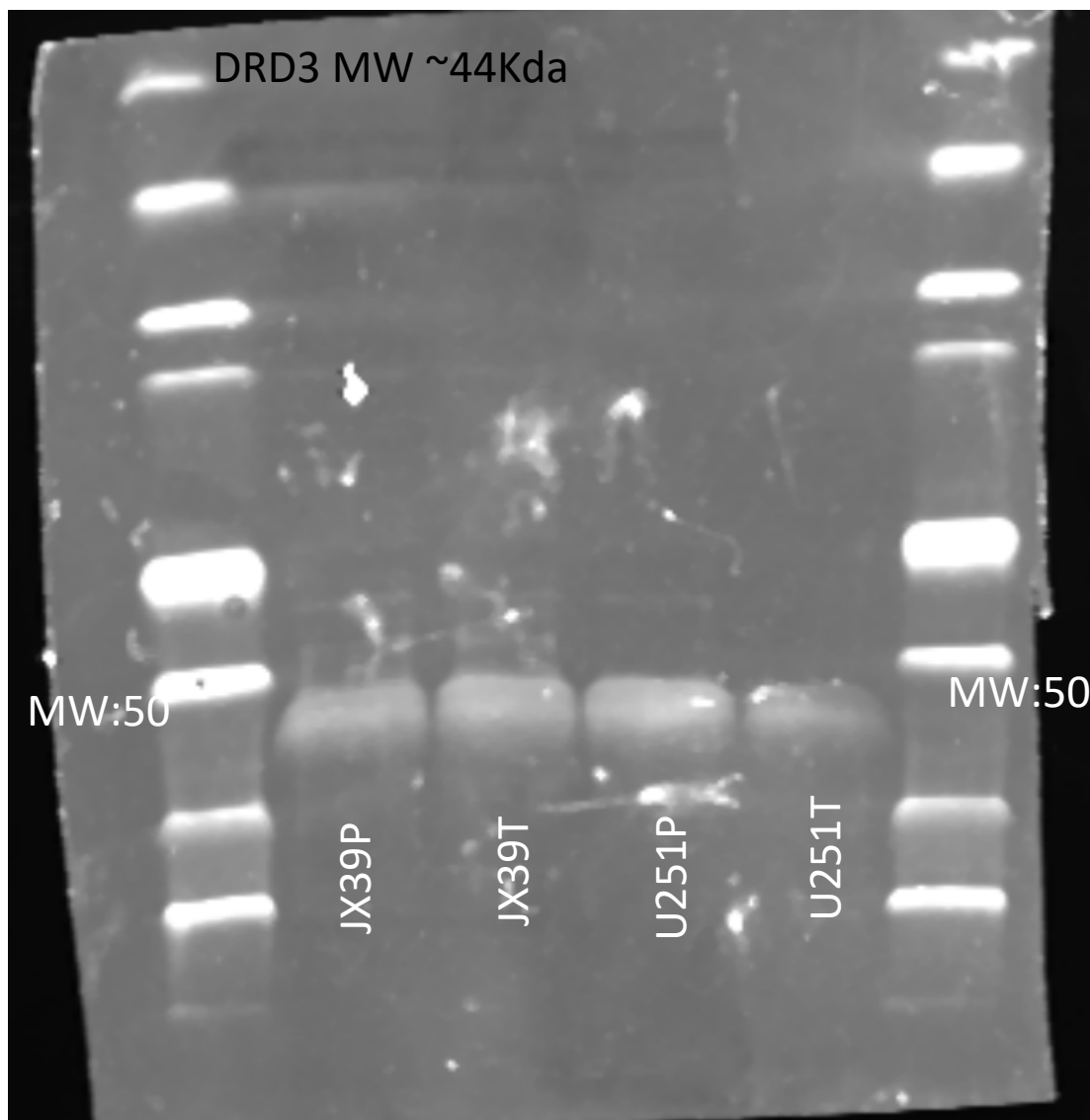

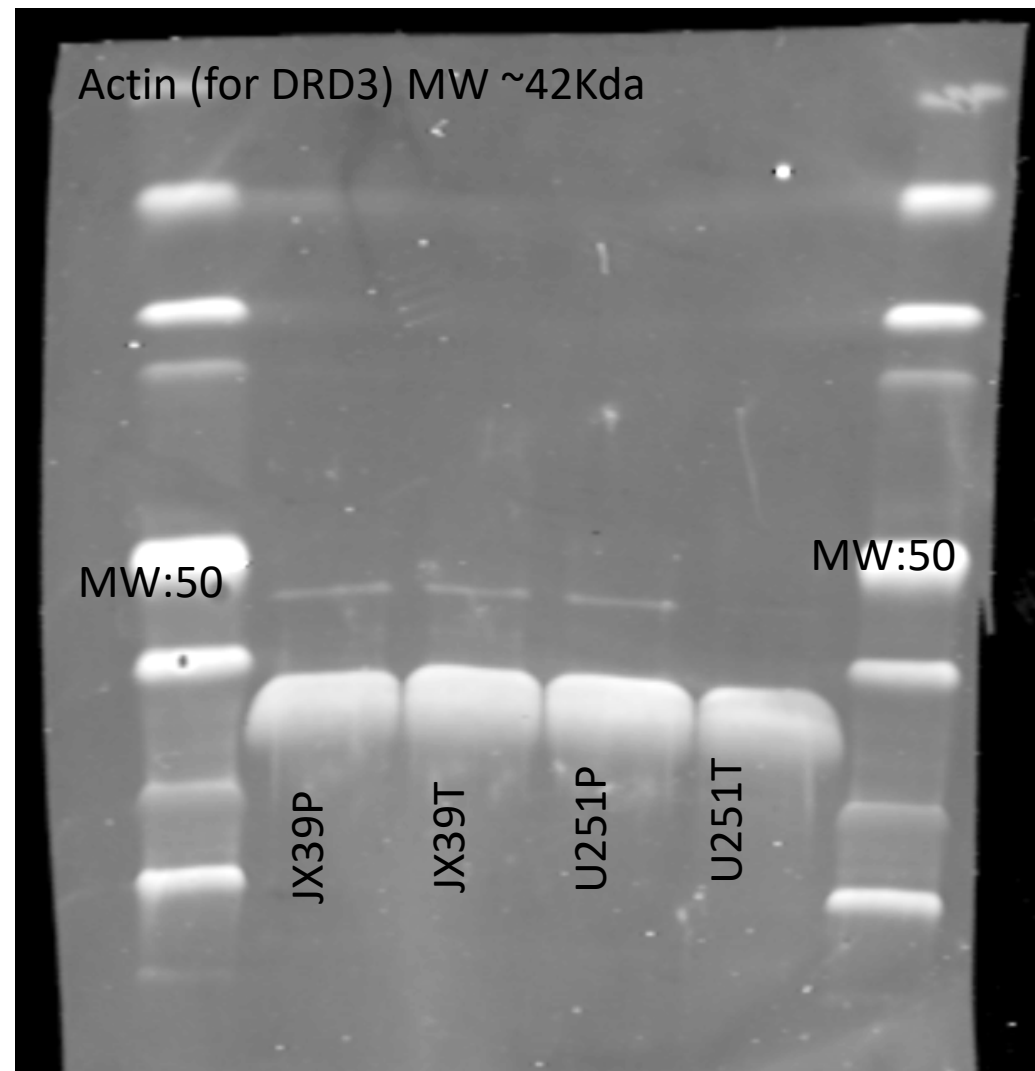

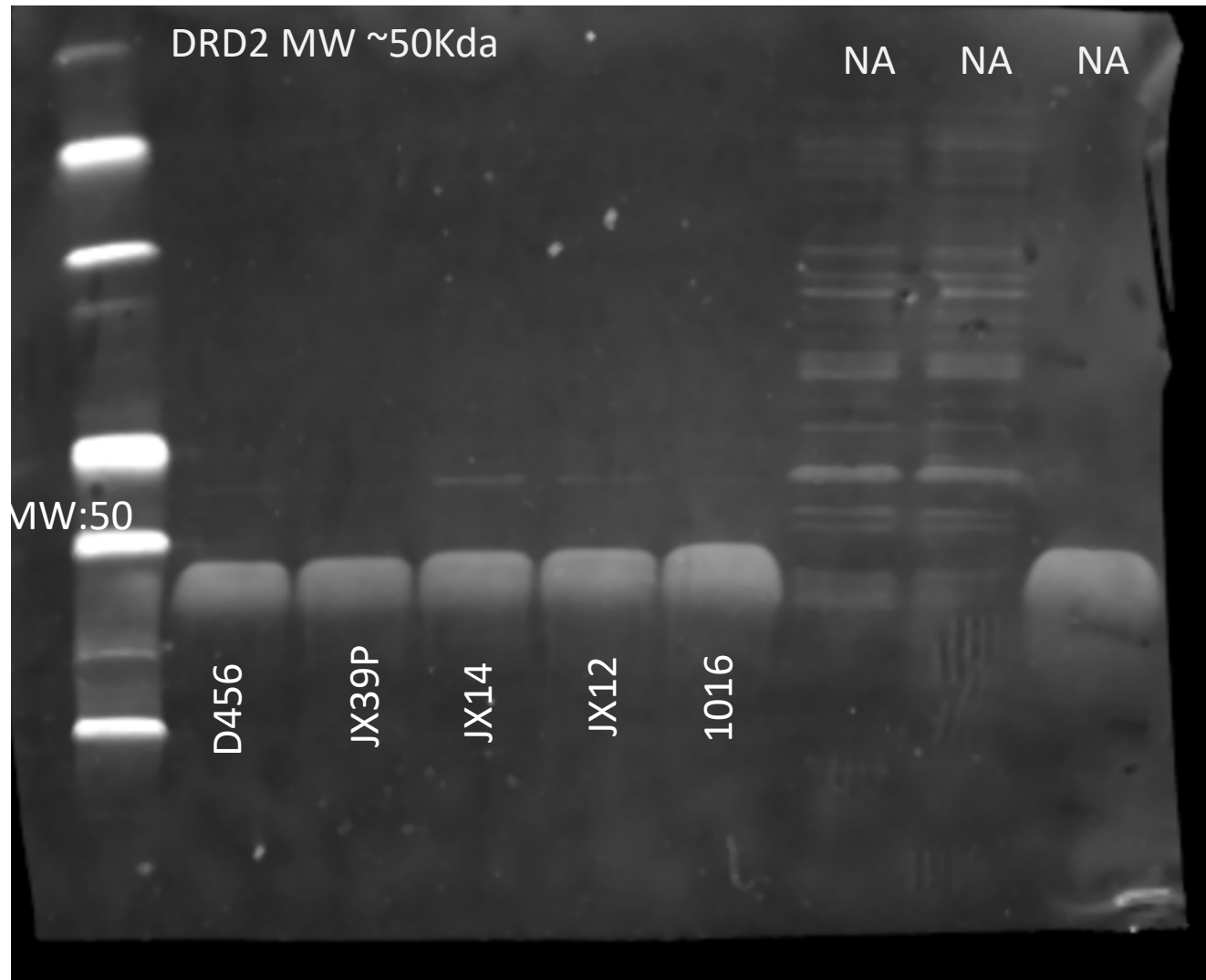

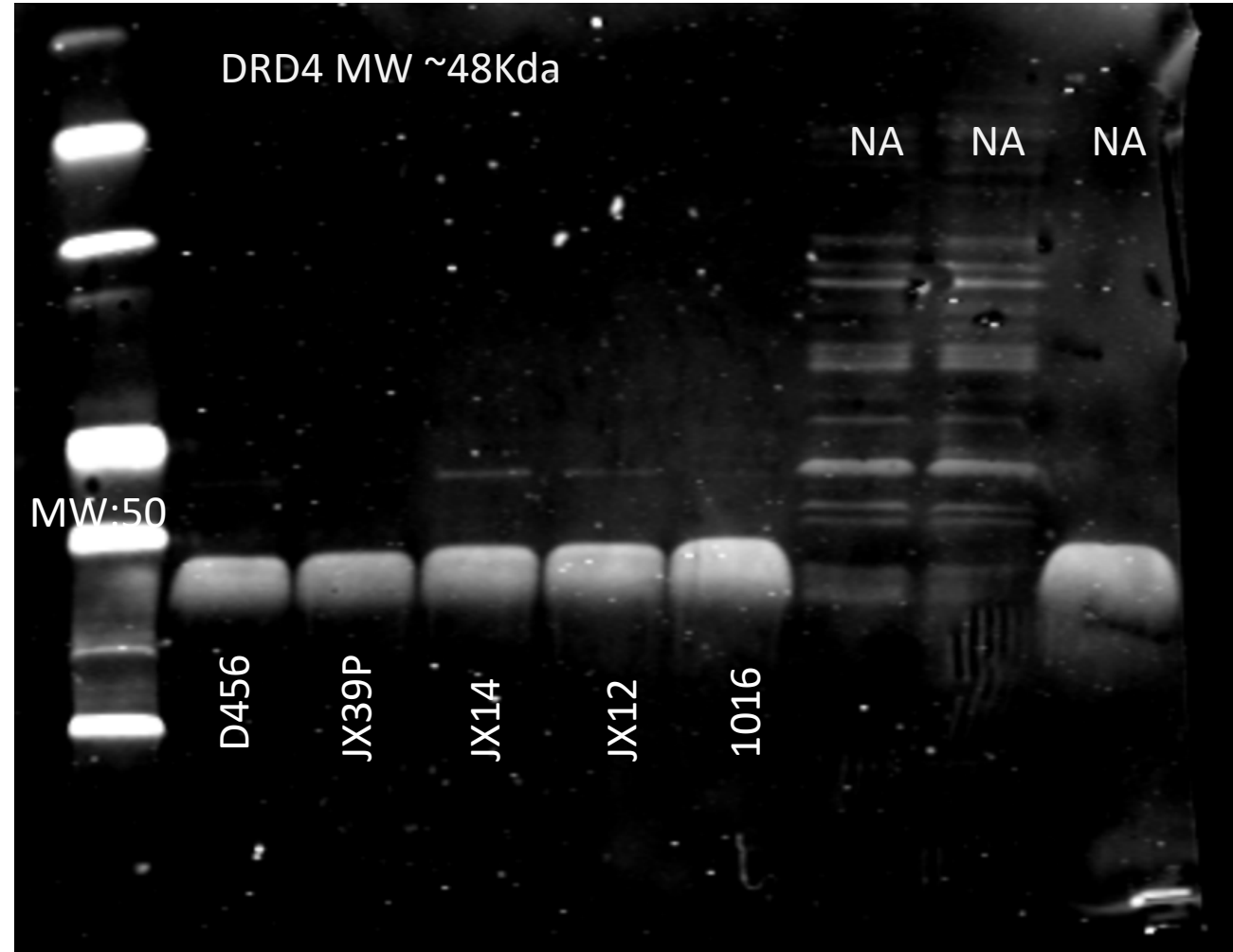

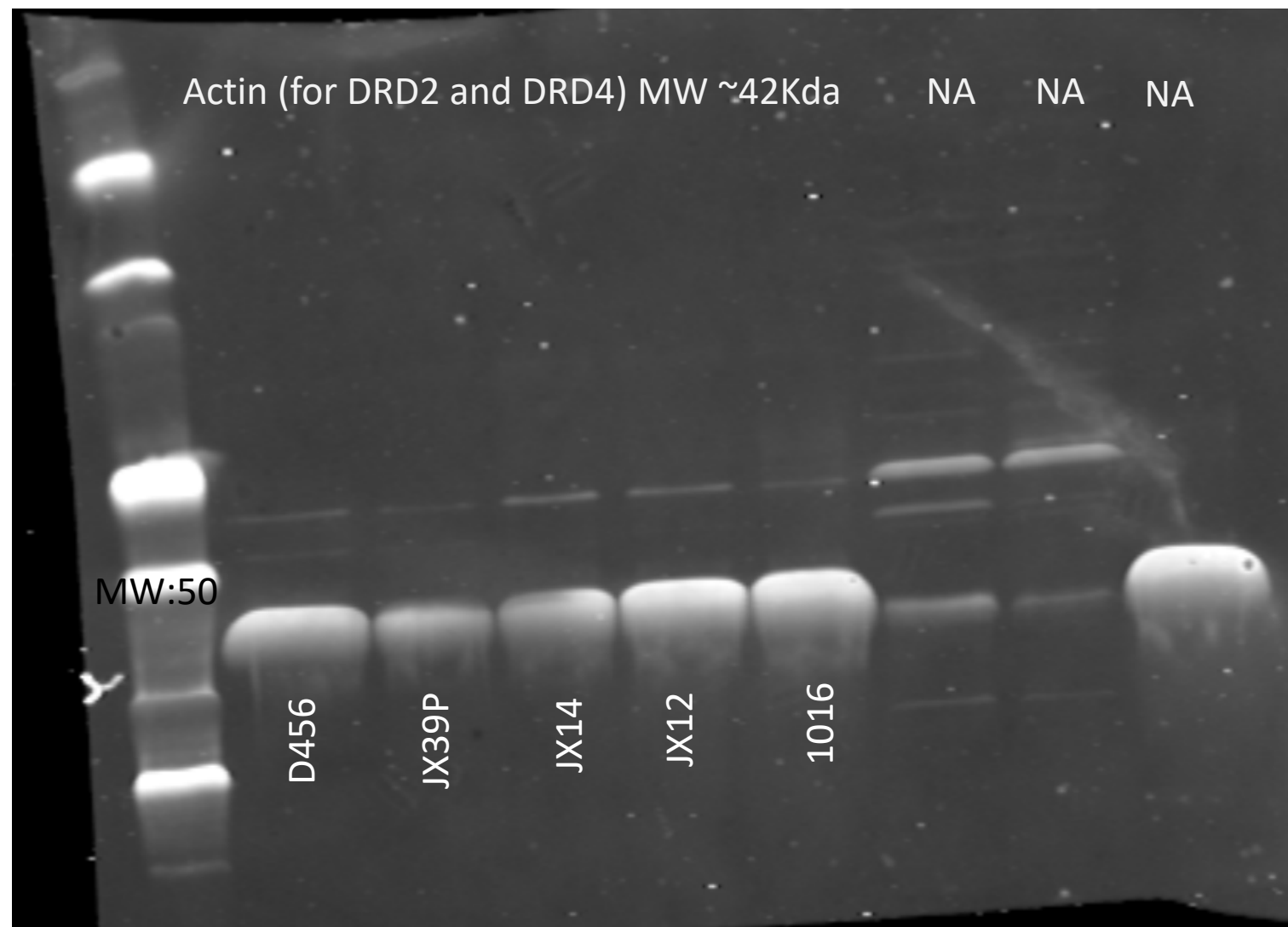

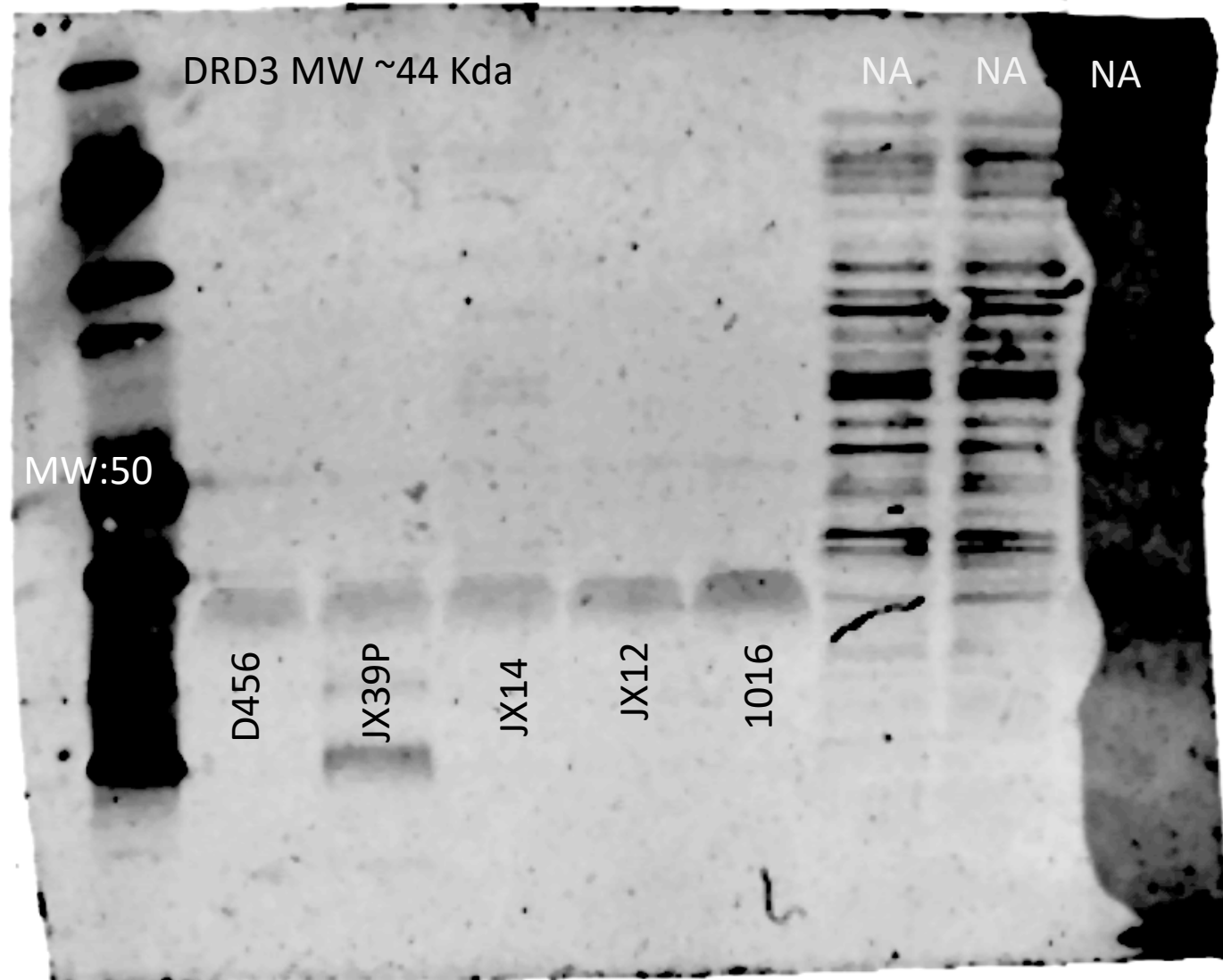

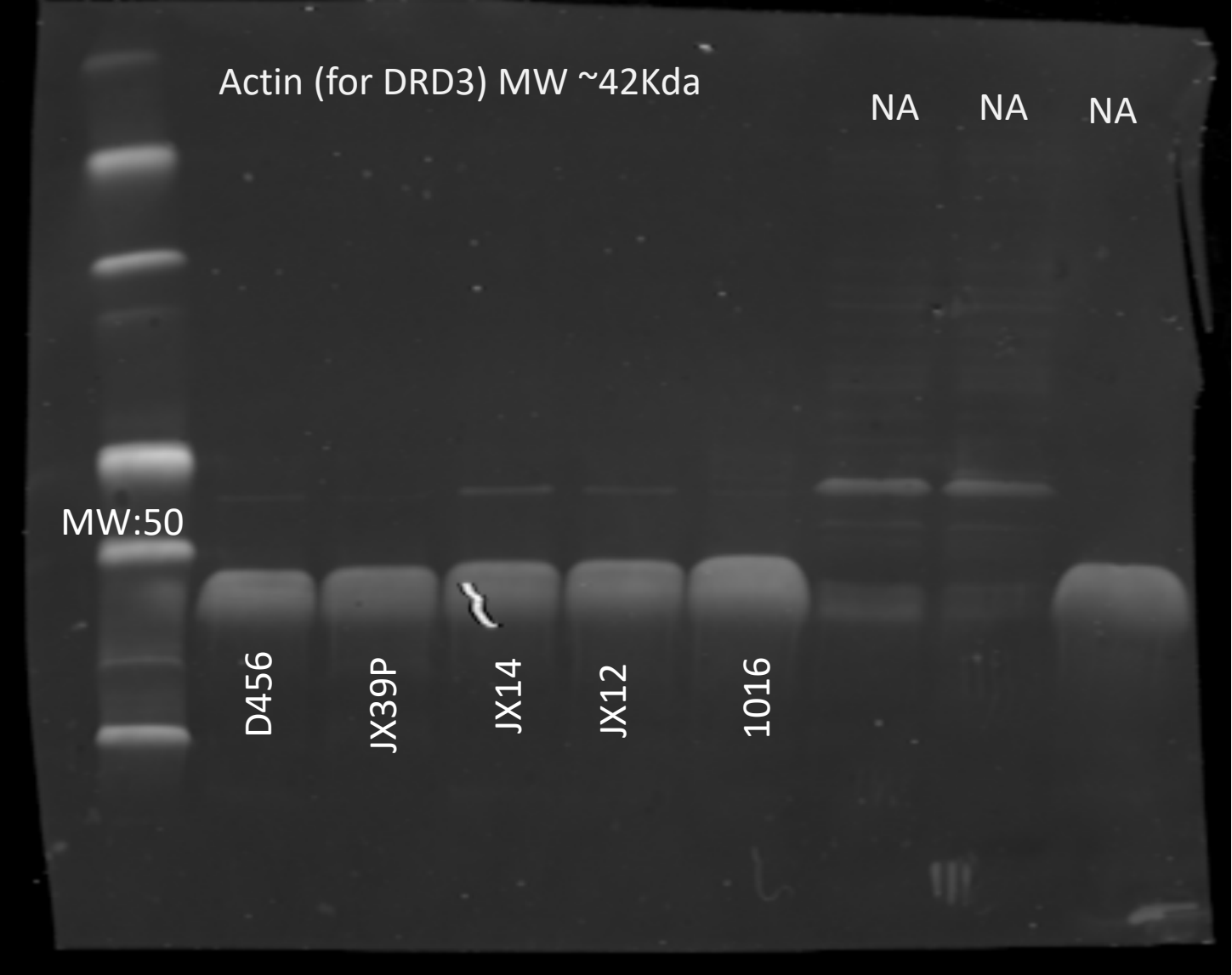

Supplement: S1 Western blots — (PDF) [file pone.0250649.s006.pdf]
